# BloodContourNet: A YOLO-Based Density-Aware Bézier Refinement Framework for Peripheral Blood Cell Detection

**DOI:** 10.3390/diagnostics16152362

**Published:** 2026-07-27

**Authors:** Mustafa Yurdakul, Eda Dönmez, Merve Ersoy, Faruk Özger, Ishak Pacal

**Affiliations:** 1 Department of Computer Engineering, Faculty of Engineering and Natural Sciences, Kırıkkale University, 71000 Kırıkkale, Türkiye; mustafayurdakul@kku.edu.tr; 2 Department of Electrical and Electronics Engineering, Faculty of Engineering and Natural Sciences, Kırıkkale University, 71000 Kırıkkale, Türkiye; 3 Department of Software Engineering, Faculty of Engineering and Natural Sciences, Istanbul Topkapı University, 34000 Istanbul, Türkiye; merveersoy@topkapi.edu.tr; 4 Department of Computer Engineering, Faculty of Engineering, Iğdır University, 76000 Iğdır, Türkiye; ishak.pacal@igdir.edu.tr; 5 Department of Computer Engineering, Faculty of Engineering and Natural Sciences, Fenerbahce University, 34758 Istanbul, Türkiye; 6 Department of Electronics and Information Technologies, Faculty of Architecture and Engineering, Nakhchivan State University, Nakhchivan 7012 AZ, Azerbaijan

**Keywords:** peripheral blood cell detection, object detection, YOLO, attention mechanism, Bézier contour refinement, density-aware reweighting, statistical validation, TXL-PBC, Bézier curve

## Abstract

**Background/Objectives:** Accurate peripheral blood cell detection remains difficult in dense smear fields, where touching erythrocytes, small platelets, and staining variability reduce the reliability of box-based object detectors. This study aims to address these limitations by proposing BloodContourNet, a contour-informed and density-aware detection framework for red blood cell, white blood cell, and platelet localization. **Method:** BloodContourNet is built on YOLOv26-L and integrates three complementary modules. The Hematological Attention Module (HAM) refines high-level features using local convolution and shifted-window attention. The Density-Aware Reweighting Head (DARH) modulates the classification loss for locally sparse or difficult instances without image-level resampling. The Bézier Contour Refinement Head (BCRH) provides a weakly supervised geometric overlap cue for ambiguous dense-region post-processing. **Results:** Experiments on the TXL-PBC dataset, containing 1260 peripheral smear images and 18,143 annotated cells, benchmarked 25 YOLO variants and 10 Transformer-based or reference detectors under a unified split and evaluation protocol. Across five random seeds, BloodContourNet achieved 99.1% mean Average Precision (mAP) at 50% Intersection-over-Union (IoU) and 87.8% mAP@50:95, improving strict-IoU performance by 1.4 points over YOLOv26-L and 0.7 points over DINO-DETR. Both margins were statistically significant across the five seeds (paired two-sided *t*-tests, both *p* < 0.05), and the five-seed 95% confidence interval of the proposed model was [87.5, 88.0]. **Conclusions:** BloodContourNet primarily improved the strict-localization setting on TXL-PBC, where dense erythrocyte regions and small platelet instances remain challenging for box-based detectors. External patient- or slide-independent validation remains necessary before broader clinical claims can be made.

## 1. Introduction

Peripheral blood smear examination remains one of the most widely used microscopic procedures for assessing erythrocytes, leukocytes, and platelets, which, respectively, support oxygen transport, immune defense, and hemostasis [1]. Accurate localization and enumeration of these cell populations provide the basis for quantitative hematological assessment and for subsequent morphology-oriented analysis [2,3]. Although manual smear review by trained specialists is still central to laboratory practice, it is labor-intensive, time-consuming, and susceptible to workload, fatigue, and inter-observer variability [4]. These constraints have increased interest in automated smear-analysis systems that can support routine microscopic review, provided that their performance is evaluated under transparent and reproducible experimental conditions.

Recent deep-learning-based approaches have substantially improved the analysis of microscopic blood images [5]. In hematology, deep models have been used for screening, classification, and quantification tasks, including B-cell acute lymphoblastic leukemia screening from peripheral smears [6], attention-augmented childhood blood-cancer detection [7], and cell-classification systems trained on public peripheral-blood resources such as PBC [8] and Raabin-WBC [9]. Other convolutional neural network-based studies have addressed automatic blood cell identification and counting [10], white blood cell recognition [11], and leukocyte-subtype classification [12]. These studies show that learned visual representations can capture relevant cytological morphology. However, many of them focus on image-level classification or category recognition, whereas practical smear analysis also requires the simultaneous detection and localization of every visible cell in a field of view, a requirement that recent YOLO-based blood cell detectors such as BC-YOLO [13] have begun to address.

Before deep detectors became dominant, automated smear-analysis pipelines commonly relied on thresholding, watershed segmentation, morphological filtering, and hand-crafted descriptors combined with shallow classifiers [14,15]. These pipelines can perform well under controlled imaging conditions, but they are sensitive to staining variability, illumination changes, cell overlap, and dense cellular arrangements. Deep object detectors have therefore become widely used for cell localization, including two-stage architectures such as Faster R-CNN [16] and Cascade R-CNN [17], single-stage YOLO detectors [18,19,20,21,22], and Transformer-based detectors such as DETR [23], Deformable DETR [24], DINO-DETR [25], and RT-DETR [26]. Transformer-based visual models have also been increasingly adopted in medical-imaging applications [27], including lightweight convolutional and Vision Transformer architectures for brain tumor classification [28,29,30] and gated, attention-driven networks for ischemic stroke lesion segmentation [31]. Single-shot YOLO detectors have likewise been used in clinical imaging beyond hematology, for example, for dental anatomy detection [32] and for the detection of interproximal, occlusal, and secondary caries on bite-wing radiographs [33]. Despite this progress, peripheral blood smear detection remains technically difficult because many erythrocytes are visually similar and often touch each other, platelets are small and under-represented, and staining or acquisition differences can alter local appearance.

These properties expose three limitations of generic box-based detectors in peripheral smear images. First, axis-aligned bounding boxes and box-Intersection-over-Union-based suppression can merge adjacent erythrocytes in crowded regions. Second, uniform classification losses may under-emphasize rare platelets, especially when platelets account for only a small fraction of all annotations [8,9]. Third, models trained on a single appearance distribution may become less reliable when staining intensity, illumination, or optical conditions shift [14,15]. Generative augmentation has been proposed as one way to reduce imbalance and appearance variability [34], but image-level oversampling can also alter the empirical class frequencies that a deployed detector is expected to encounter. A more targeted detector should therefore improve appearance robustness, adjust optimization for locally sparse or difficult instances, and refine dense-region localization without requiring additional mask annotations.

Contour-aware representations offer a possible way to improve localization when rectangular boxes are insufficient. Bézier curves have been used for arbitrary-shaped scene-text detection in ABCNet [35], while boundary-, polygon-, and snake-based representations have been explored in SharpContour [36], PolyTransform [37], and DeepSnake [38]. Boundary-aware modeling has also been emphasized in medical image segmentation [39]. Most of these approaches, however, rely on dense pixel-level supervision and are designed for segmentation rather than box-supervised object detection. Public peripheral blood smear datasets are commonly released with bounding-box labels only, which limits the direct use of fully supervised contour or mask heads. This motivates a weaker formulation in which a compact contour representation is used not as a verified biological boundary, but as an auxiliary geometric cue for separating ambiguous detections in dense smear regions.

The present study addresses this gap by proposing BloodContourNet, a YOLOv26-L-based framework for peripheral blood cell detection. YOLOv26-L was selected as the backbone because it provided the strongest strict-localization performance among the evaluated YOLO variants in our benchmark while preserving real-time inference. The framework integrates three modules. The Hematological Attention Module (HAM) refines high-level features using local convolution and shifted-window attention to improve representation under appearance variability. The Density-Aware Reweighting Head (DARH) modulates the classification loss according to local instance density and difficulty without image-level resampling. The Bézier Contour Refinement Head (BCRH) predicts a weakly supervised geometric contour representation that supplies an auxiliary overlap cue during ambiguous dense-region post-processing.

All experiments were conducted on TXL-PBC [40], a public peripheral blood cell detection benchmark containing red blood cell, white blood cell, and platelet annotations. We also explicitly acknowledge that TXL-PBC does not provide patient- or slide-level identifiers; therefore, the reported results should be interpreted as within-dataset performance rather than evidence of patient-independent or source-independent clinical generalization. Building on our earlier blood cell detector, BC-YOLO [13], the present work shifts the focus from coarse-IoU detection toward strict-IoU localization, platelet detection, and dense-cluster separation. The main contributions of this study are as follows:

• We propose BloodContourNet, a contour-informed and density-aware extension of YOLOv26-L for strict-Intersection-over-Union peripheral blood cell detection, with particular focus on dense erythrocyte regions, small platelets, and appearance variability.
• A weakly supervised Bézier Contour Refinement Head is introduced to provide an auxiliary contour-based overlap cue from box-derived geometric priors and teacher consistency, enabling dense-region post-processing without pixel-level mask annotations.
• The framework integrates a Hematological Attention Module and a Density-Aware Reweighting Head to refine high-level features under staining variability and to modulate classification learning for locally sparse or difficult instances.
• A broad controlled benchmark is conducted on TXL-PBC, including 25 YOLO variants and 10 Transformer-based or reference detectors evaluated under a unified split, metric definition, and evaluation protocol.
• The evaluation includes five-seed experiments, confidence intervals, paired statistical tests, class-level and platelet-specific analyses, computational efficiency assessment, and sensitivity studies for the contour and loss-weight parameters.

The remainder of this paper is organized as follows. Section 2 describes the dataset, leakage assessment, baseline detectors, and the proposed BloodContourNet framework. Section 3 presents the experimental setup, evaluation metrics, and statistical protocol. Section 4 reports quantitative comparisons, ablation results, class-level analysis, efficiency analysis, sensitivity experiments, qualitative findings, and training behavior. Section 5 discusses the implications and limitations of the results, and Section 6 concludes the study.

## 2. Materials and Methods

### 2.1. Dataset

**Figure 1:**
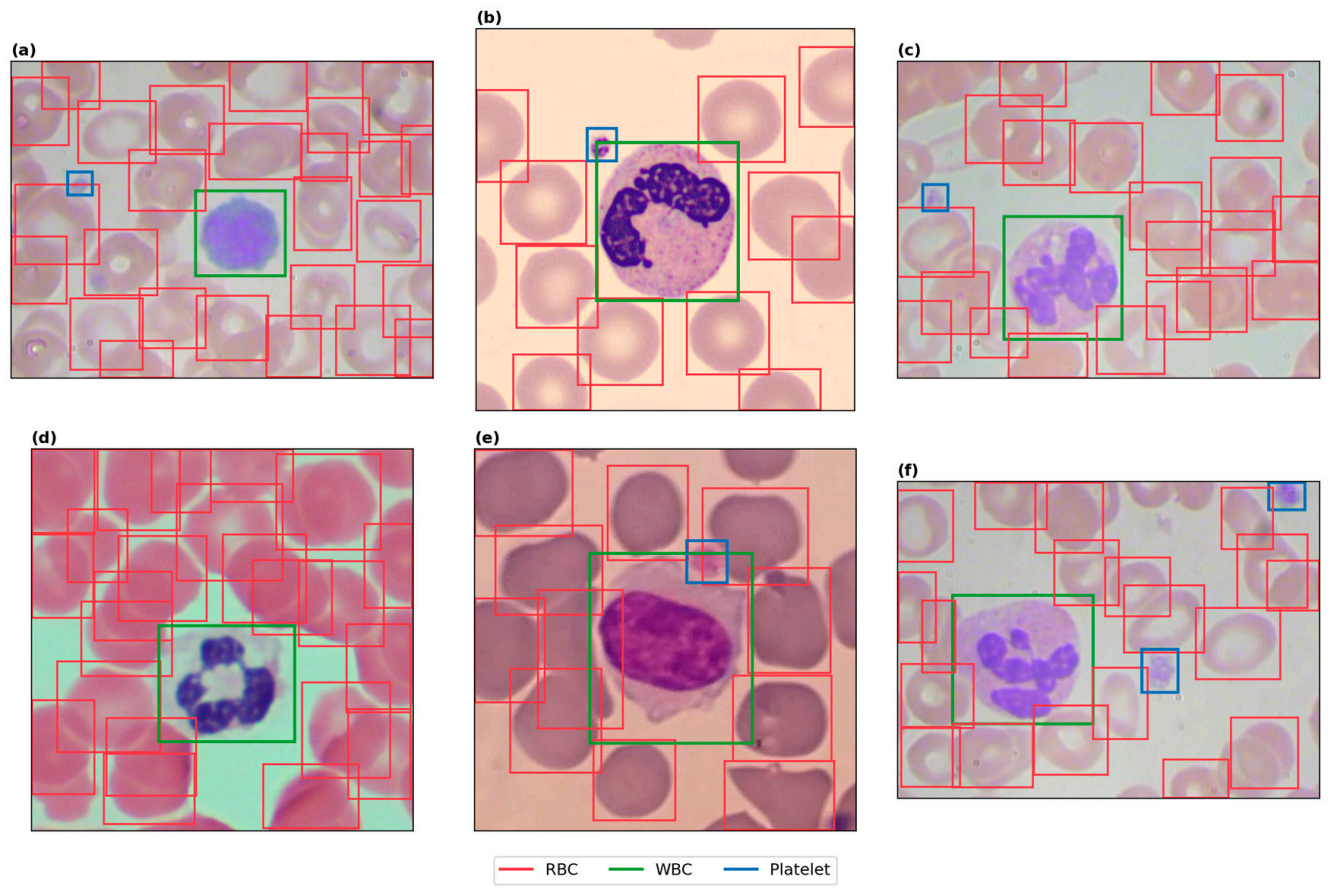
Six representative test images (**a**–**f**) from the TXL-PBC dataset with the original ground-truth bounding boxes overlaid. Red boxes denote erythrocytes (RBC), green boxes denote leukocytes (WBC), and blue boxes denote platelets. (**a**) Densely populated field with light, neutral staining, one leukocyte, and a single platelet. (**b**) Sparsest field, with pronounced pink staining and an unusually platelet-rich background, containing one leukocyte and numerous small platelet annotations. (**c**) Moderately populated field with light staining, one leukocyte, and a single platelet. (**d**) Most crowded field, showing extensive erythrocyte overlap under darker staining, with one leukocyte and several platelets. (**e**) Moderately populated field with the most intense staining and the lowest background brightness, containing one leukocyte and a single platelet. (**f**) Moderately populated field with neutral staining, one leukocyte, and two platelets. The panels illustrate the variation in cell density, staining intensity, and field-of-view that characterizes the dataset.

All experiments were conducted on TXL-PBC [40], a public peripheral blood cell detection dataset containing 1260 microscopic smear images with bounding-box annotations. The dataset includes 18,143 annotated cell instances from three classes: 16,302 red blood cells, 1298 white blood cells, and 543 platelets. The class distribution is strongly imbalanced, with red blood cells accounting for 89.9% of all annotations, white blood cells for 7.2%, and platelets for 3.0%. The original image distribution was retained throughout the experiments, and no image-level oversampling was applied. Class imbalance was handled only through the loss-level mechanism described in Section 2.6. The dataset was divided into training, validation, and test subsets using a 7:2:1 ratio, corresponding to 882, 252, and 126 images, respectively (Table 1). Representative test images with ground-truth annotations are shown in Figure 1. TXL-PBC does not provide per-image acquisition metadata. Among the source descriptors, Raabin-WBC images were acquired at 100× magnification [9] and BCDD images with a high-magnification light microscope, whereas PBC (CellaVision DM96 analyser) and BCCD report no numerical objective magnification. All images were letter-boxed to 640 × 640, so no pixel-to-micrometre calibration is available and all measurements are in pixel units. 

The 7:2:1 partition was generated as a fixed image-level split using seed 0, and the same split files were used for every detector in the benchmark. This split was not inherited from the TXL-PBC descriptor [40], which reports results under its own experimental protocol. It was selected to provide a conventional 70/20/10 training, validation, and test division while preserving a sufficiently large validation subset for hyperparameter and loss-weight selection. Because TXL-PBC does not provide patient- or slide-level identifiers, patient-grouped or slide-grouped splitting was not possible. The test results should therefore be interpreted as held-out image-level performance on TXL-PBC rather than as a patient-independent or slide-independent generalization.

### 2.2. Data Leakage Assessment

TXL-PBC integrates four public resources [8,9,41,42], but the released annotations do not include patient-level or slide-level identifiers. As a result, patient-grouped and slide-grouped splitting could not be performed, and potential same-source or same-slide correlation cannot be excluded solely from the metadata. To assess the risk of near-duplicate leakage, each test image was compared with every training image using three complementary measures: 64-bit perceptual hash Hamming distance, cosine similarity of ImageNet-pretrained ResNet-50 global features, and the structural similarity index. For each test image, the closest training image was retained as the worst-case match. No test image met the predefined near-duplicate criteria, namely a pHash Hamming distance ≤ 5, feature cosine similarity ≥ 0.97, or structural similarity index ≥ 0.90. These thresholds were chosen conservatively so that the screen would over-flag rather than miss potential duplicates. A Hamming distance of 5 over the 64 hash bits corresponds to agreement in more than 92% of the bits, a level at which two images are visually near-identical, whereas unrelated smear fields typically differ in many more bits. A feature cosine similarity of 0.97 lies close to unity and is appropriate because deep global descriptors of same-modality microscopy images are already strongly correlated; therefore, only near-identical content exceeds it. However, a structural similarity index of 0.90 marks pairs whose pixel-level luminance, contrast, and structure are almost the same. Because the three criteria capture complementary aspects of similarity—global appearance, semantic feature content, and pixel-level structure—a test image was treated as a potential near-duplicate whenever its perceptual-hash distance fell to or below the distance threshold, or either similarity score reached its threshold. The maximum feature cosine similarity was 0.91, the maximum structural similarity index was 0.78, and the mean nearest-training-image cosine similarity was 0.62. This analysis reduces the likelihood of exact or near-exact duplication between training and test partitions, but it cannot rule out same-patient, same-slide, or same-source correlation in the absence of identifiers. The reported test performance should therefore be read as image-level held-out performance on TXL-PBC.

### 2.3. Baseline Detectors

The proposed model was compared with single-stage, query-based, and reference object detectors under the same TXL-PBC test partition and metric definitions. The YOLO group included YOLOv9, YOLOv10, YOLOv11, YOLOv12, and YOLOv26 variants [18,19,20,21,22] across multiple model scales. The Transformer- and query-based groups included DETR, Deformable DETR, DINO-DETR, RT-DETR-L, and RT-DETR-X [23,24,25,26]. Faster R-CNN, Cascade R-CNN, FCOS, CenterNet, and EfficientDet-D4 [16,17,43,44,45] were included as additional reference detectors. This design provides a broad comparison against real-time single-stage models, query-based detectors, and refinement-oriented architectures while keeping the test set and evaluation metrics fixed.

### 2.4. Proposed Framework: BloodContourNet

**Figure 2:**
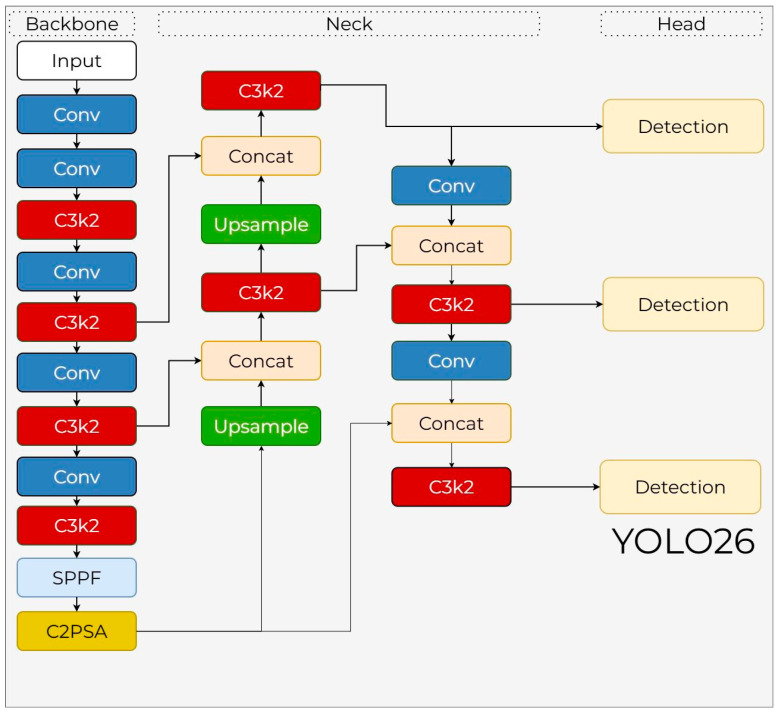
Architecture of the YOLOv26 detector used as the backbone of BloodContourNet. The backbone applies successive Conv and C3k2 blocks followed by an SPPF module and a C2PSA cross-stage partial attention block to produce multi-scale features. The neck follows a PAN-FPN design, fusing features through Upsample and Concat operations along top-down and bottom-up paths built from C3k2 and Conv blocks. The three resulting feature scales are passed to independent detection heads, on top of which the proposed HAM, DARH, and BCRH modules are integrated.

**Figure 3:**
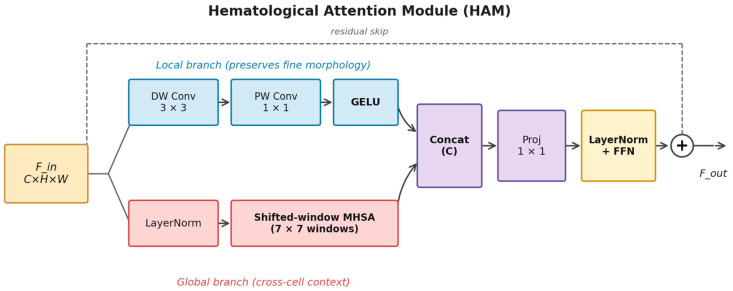
Internal structure of the Hematological Attention Module (HAM). The local depth-wise convolutional branch and the shifted-window multi-head self-attention branch operate in parallel on the input feature map. Their outputs are concatenated, projected, and refined by a LayerNorm + FFN block, and then added to the input through a residual connection.

**Figure 4:**
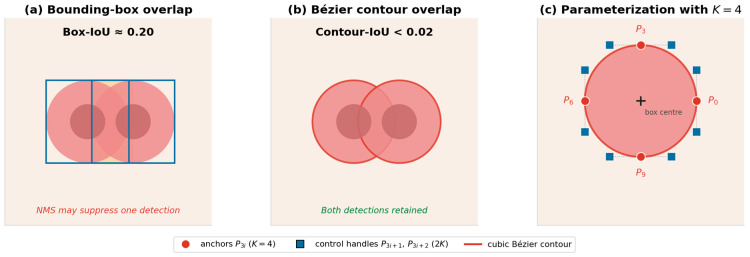
Bézier Contour Refinement Head (BCRH): (**a**) Two touching circular cells enclosed by axis-aligned boxes with IoU = 0.20. This schematic example is intended only to illustrate how rectangular boxes can overestimate the spatial interaction between adjacent rounded cells; it is not the operational threshold used to trigger contour-informed post-processing. (**b**) The same pair represented by closed cubic Bézier contours; the contour-based overlap remains lower than the box-level overlap, showing why a contour cue can provide additional geometric information for ambiguous dense-region decisions. (**c**) Parameterization with K = 4 anchor points and 2K control points regressed by the auxiliary head.

BloodContourNet was designed around three difficulties commonly encountered in peripheral smear detection: separation of touching erythrocytes, recovery of small platelet instances, and robustness to staining or acquisition variability. The framework uses YOLOv26-L as the base detector and adds three task-specific components. The Hematological Attention Module (HAM) refines the deepest semantic features through local convolution and shifted-window attention. The Density-Aware Reweighting Head (DARH) modifies only the classification component of the detection loss using local instance density. The Bézier Contour Refinement Head (BCRH) predicts a compact Bézier-based geometric representation, used as an auxiliary overlap cue during ambiguous dense-region post-processing. The three modules are optimized jointly with the standard detection losses using λ_1_ = 7.5, λ_2_ = 1.5, λ_3_ = 2.0, and λ_4_ = 0.5. The official evaluation remains based on bounding boxes, preserving direct comparability with all baseline detectors. The backbone architecture is shown in Figure 2, the internal structure of HAM in Figure 3, and the contour parameterization of BCRH in Figure 4.

### 2.5. Hematological Attention Module (HAM)

HAM refines the deepest backbone feature map $F\in R^{C\times H\times W}\;$through two parallel branches followed by residual fusion (Figure 3). The first branch is a local convolutional path composed of a 3×3 depth-wise convolution and a 1×1 point-wise projection. This branch emphasizes local texture, edge-like patterns, and small spatial variations that are relevant for cell appearance, without making explicit claims about biological boundary recovery. The second branch applies shifted-window multi-head self-attention within non-overlapping 7×7 windows, with a half-window cyclic shift between consecutive applications. This design captures neighborhood context across nearby cells while avoiding the computational cost of global self-attention. The two branch outputs are concatenated along the channel dimension, projected back to C channels, refined by a LayerNorm and feed-forward block, and added to the input through a residual connection. The windowed attention reduces the complexity from $O(\left(HW{)}^{2}C\right)\;$to $O(HW\cdot M^{2}\cdot C)$, where M=7 denotes the window side length. HAM adds approximately 1.1 M parameters and 2.9 GFLOPs at the deepest stage, and its placement at the lowest-resolution feature level keeps the additional cost limited relative to the full detector. HAM is not used as a full-network feature extractor but as a single lightweight refinement block applied only to the deepest, lowest-resolution feature map, unlike general-purpose attention modules such as CBAM [46] that are inserted broadly across a network. It reuses the shifted-window mechanism of the Swin Transformer [47] only as one of two parallel branches rather than as a whole-backbone attention hierarchy. Its specific contribution is the parallel combination of a local depth-wise convolutional path, which preserves fine cell-texture and edge cues, with a shifted-window attention path that models context across neighboring cells, fusing both within one residual block to target the appearance variability of stained smears while adding only about 1.1 M parameters. This differs from distributing attention throughout the backbone, which raises the cost without specifically addressing the deepest-stage semantic features most affected by staining and illumination changes.

### 2.6. Density-Aware Reweighting Head (DARH)

DARH introduces a local-density classification weight for each annotated instance. Its purpose is not to estimate global class frequency or to resample platelet-rich images, but to adjust the classification contribution of each object according to its local spatial context. For each annotated instance i, the local density score is defined as the number of neighboring ground-truth boxes whose centers fall within a scale-adaptive radius around that instance (Equation (1)):

(1) $$d_{i}=\sum_{j\neq i}1\left[∥c_{i}-c_{j}{∥}_{2}\;\leq \;\rho s_{i}\right],$$

where $c_{i}\;$denotes the center of box i, $s_{i}=\sqrt{w_{i}\cdot h_{i}}$ denotes its geometric scale, ρ=1.5 is the neighborhood factor, and 1[⋅] is the indicator function. The resulting score $d_{i}\;$is a local crowding measure and should not be interpreted as a global class-imbalance statistic. To reduce mini-batch fluctuation, a running density mean is updated with an exponential moving average at each training step (Equation (2)):

(2) $$\mu_{d}^{\left(t\right)}=m\mu_{d}^{\left(t1\right)}+\left(1-m\right)\frac{1}{B}\sum_{i=1}^{B}d_{i},$$

where B is the number of annotated instances in the mini-batch, and m=0.99 is the EMA momentum. The classification weight is then obtained by comparing the instance density with the running mean through a logistic gate (Equation (3)):

(3) $$w_{i}=\sigma \left(a(\mu_{d}-d_{i})+b\right),$$

where σ is the logistic sigmoid, and a and b are learnable scalars initialized to 1 and 0, respectively. Instances with lower local density receive larger classification weights, which emphasizes locally isolated or easily overlooked cells without duplicating images or changing the empirical class distribution. The weight $w_{i}$ is applied only to the classification term of the detection loss. Bounding-box regression and Distribution Focal Loss remain unchanged, so geometric supervision is not weakened for crowded regions. In this formulation, DARH should be interpreted as a local-density classification reweighting mechanism rather than a general hard-example mining module. This distinguishes DARH from the two families of imbalance-handling techniques most common in detection. Unlike focal loss [48], which reweights every prediction by its classification confidence as a proxy for difficulty and ignores spatial arrangement, DARH derives its weight from the local geometric crowding of each ground-truth instance; therefore, locally isolated cells are emphasized even when they are already confidently classified. Unlike online hard-example mining [49], which trains on a selected subset of high-loss samples, DARH keeps every sample and neither duplicates nor discards images; therefore, the empirical class frequencies of the training set are left unchanged—an important property when platelets constitute only 3.0% of the annotations. Its contribution is thus a purely local, distribution-preserving reweighting signal rather than a global class-frequency or gradient-hardness adjustment.

### 2.7. Bézier Contour Refinement Head (BCRH)

BCRH predicts a compact closed Bézier representation for each candidate cell. The representation is used as a geometric overlap proxy rather than as a mask-supervised cell boundary. Two design questions motivate this head: why a contour cue is introduced at all, and why the contour is represented with Bézier curves rather than an alternative boundary formulation. Axis-aligned box-IoU systematically overestimates the overlap of two adjacent rounded cells because the empty corners of each box inflate the intersection, so a shape-aware overlap measure is useful precisely in the dense touching-erythrocyte regions where box-level suppression tends to merge neighbors. Among shape representations, cubic Bézier curves are particularly well suited to this setting: blood cells are approximately round or elliptical with smooth boundaries, and a closed cubic Bézier contour reproduces such shapes with very few parameters—here, four anchors and eight control points—which keeps the auxiliary head lightweight and preserves real-time inference. The same real-time Bézier parameterisation was introduced for arbitrary-shaped scene text in ABCNet [35], although there it serves as a fully supervised detection output rather than an auxiliary cue. By comparison, pixel-mask or segmentation-style boundary heads such as SharpContour [36] require dense per-pixel supervision, which TXL-PBC does not provide. Polygon-based representations such as PolyTransform [37] instead need many vertices to appear smooth and can leave jagged boundaries at low vertex counts. Active-contour or snake-based methods such as DeepSnake [38] deform an initial contour iteratively and are heavier and more sensitive to initialisation, whereas a Bézier curve is regressed in a single closed form and yields an inherently smooth, analytically defined boundary that supports a cheap, well-defined contour-IoU computation between neighboring detections. BCRH therefore repurposes this compact contour representation as a weakly supervised geometric overlap proxy for disambiguating dense candidate pairs during post-processing, which is, to our knowledge, a use of Bézier contours not previously reported for peripheral blood cell detection. The contour is parameterized by K anchor points and 2K control points, and each cubic segment is evaluated as shown in Equation (4):

(4) $$B_{i}(t)=(1-t{)}^{3}P_{3i}+3(1-t{)}^{2}tP_{3i+1}+3(1-t)t^{2}P_{3i+2}+t^{3}P_{\left(3i+3\right)\;mod\;3K},$$

where t∈[0,1], P denotes the anchor or control points, and the modulo indexing ensures that adjacent segments share anchors and form a closed $C^{0}$-continuous curve. We set K=4 to keep the branch lightweight while preserving enough flexibility for approximately rounded cell shapes. The sensitivity of accuracy and speed to K is analyzed in Section 4.7. The head regresses the $\left(\Delta x\Delta y\right)$ displacement of each control point relative to the predicted box center, normalized by the box diagonal to reduce scale sensitivity. Figure 4 illustrates the intended use of BCRH: when adjacent cells have overlapping axis-aligned boxes, their Bézier-based overlap may remain lower, providing an auxiliary cue for ambiguous dense-region suppression.

Because TXL-PBC provides bounding boxes rather than pixel-level masks, BCRH is trained with weak geometric supervision rather than true boundary supervision. During training, the predicted Bézier curve is converted into a polygon C by sampling 64 equally spaced points along the four cubic segments. The weak target $C^{*}$ is defined as a box-aligned elliptical prior derived from the corresponding bounding box. The Bézier-IoU loss measures the overlap between the predicted polygon and this weak target (Equation (5)):

(5) $$L_{BIoU}\left(C,C^{*}\right)=1-\frac{|C\cap C^{*}|}{|C\cup C^{*}|}.$$

The target $C^{*}$ is not intended to represent the exact biological cell boundary; it provides a stable geometric anchor for the auxiliary contour branch. Increasing the number of sampled points from 64 to 128 changed the estimated polygon area by less than 0.3% on average, so 64 points were used to balance numerical stability and computational cost. To reduce unstable updates in the auxiliary Bézier branch, the Bézier-IoU term is combined with an exponential-moving-average teacher-consistency loss (Equation (6)). Since each closed Bézier contour is represented by K anchor points and 2K control points, the complete Bézier point set is defined as $P=\{P_{k}{\}}_{k=1}^{3K}$, with the EMA teacher counterpart denoted as $P^{\prime }=\{P_{k}^{\prime }{\}}_{k=1}^{3K}$. The consistency loss is therefore computed over all anchor and control points (Equation (6)):

(6) $$L_{cons}=\frac{1}{3K}\sum_{k=1}^{3K}∥P_{k}-P_{k}^{\prime }{∥}_{2}^{2}$$

where $P_{k}$ and $P_{k}^{\prime }$ denote the corresponding student and EMA teacher Bézier points, respectively. This term stabilizes the trajectory of the auxiliary contour representation during training, but it does not provide true boundary supervision. The predicted Bézier shape is therefore used only as a geometric overlap proxy for detection post-processing, not as a verified biological cell contour. The complete training objective is defined in Equation (7):

(7) $$L_{total}=\lambda_{1}L_{box}+\lambda_{2}L_{cls}^{DARH}+\lambda_{3}L_{BIoU}+\lambda_{4}L_{cons},$$

where $L_{box}$ is the standard box-regression loss, $L_{cls}^{DARH}\;$is the DARH-weighted classification loss, $L_{BIoU}$ supervises the weak Bézier overlap proxy, and $L_{cons}\;$regularizes the control-point trajectory. The validation-selected weights were $\lambda_{1}=7.5$, $\lambda_{2}=1.5$, $\lambda_{3}=2.0$, and $\lambda_{4}=0.5$. These weights were obtained through a coarse validation grid search (Section 4.6). BCRH and DARH were activated from epoch 10 onward to avoid unstable early optimization before reliable box predictions had formed.

### 2.8. Contour-Informed Post-Processing

During inference, the Bézier contour predicted by BCRH is used only after standard box-level candidate generation. YOLOv26-L and BloodContourNet use the same confidence filtering, candidate selection, and class-wise box-level suppression settings before the contour check is applied. The schematic IoU value shown in Figure 4 is used only to visualize the difference between box-level and contour-level overlap for adjacent rounded cells; it is not used as the operational trigger for contour-informed post-processing. In the actual inference pipeline, the contour check is applied only to same-class candidate pairs in dense regions whose box-IoU falls within the ambiguous range of 0.45 to 0.75. For these pairs, the sampled Bézier polygons, Ca and Cb, are used to compute contour-IoU, which is depicted in Equation (8). If the contour-IoU is lower than the validation-selected contour threshold τcont, both detections are retained despite overlapping boxes; otherwise, the standard suppression decision is preserved. Candidate pairs with a box-IoU below 0.45 are left unchanged, whereas pairs with a box-IoU above 0.75 follow the standard box-level suppression rule. This check is therefore restricted to ambiguous dense-region cases rather than being applied to all detections. Contour rasterization and polygon intersection add an average overhead of 0.6 ms per image on the test hardware, and this cost is included in the reported inference speed.

(8) $$\mathrm{Contour}\text{-}\mathrm{IoU}=\frac{Area\;(C_{a}\cap C_{b})}{Area\;(C_{a}\cup C_{b})}.$$

## 3. Experimental Setup

All experiments were conducted on a single workstation using the fixed TXL-PBC train, validation, and test split described in Section 2.1. The same test set, input resolution, confidence-thresholding policy, and metric definitions were used across the 35 baseline detectors and BloodContourNet. The hardware configuration is reported in Table 2, and the training settings used for BloodContourNet and the YOLO-family baselines are summarized in Table 3.

All models were implemented in PyTorch 2.1 with automatic mixed-precision training. YOLO-family detectors were trained with the official Ultralytics implementation, DETR-family detectors with MMDetection, and the remaining reference detectors with Detectron2. All images were resized with letter-boxing to 640×640 and normalized according to the convention of each framework. YOLO-family models followed the same base training recipe, including Mosaic, MixUp, HSV jitter, scale and translation augmentation, and horizontal flipping. DETR-family and Detectron2-based models were trained with their standard detection configurations, with the input resolution and evaluation protocol matched to the YOLO experiments.

Detector-specific training recipes were retained when required by the original implementations, but evaluation was performed under a common protocol to avoid differences in test-time scoring. The benchmark should therefore be interpreted as a controlled comparison under the same split, input size, and metric definitions rather than as proof that every architecture was optimized to its theoretical best configuration. BloodContourNet used the YOLOv26-L recipe as its base configuration, and the BCRH and DARH branches were activated from epoch 10 to reduce early optimization instability. A full training run took approximately 4.5 h for the YOLO baselines and 7.2 h for BloodContourNet.

### 3.1. Evaluation Metrics

Detection performance was assessed with standard object-detection metrics that measure prediction correctness, object recovery, localization quality, and computational cost. Precision measures the proportion of predicted objects that match ground-truth annotations, while recall measures the proportion of annotated objects recovered by the detector. These metrics are defined in Equations (9) and (10):

(9) $$Precision=\frac{TP}{TP+FP},$$

(10) $$Recall=\frac{TP}{TP+FN},$$

where TP, FP, and FN denote true positives, false positives, and false negatives, respectively. In this three-class setting, recall is especially relevant for platelets because they are small and under-represented in TXL-PBC. The dataset contains red blood cells, white blood cells, and platelets only; therefore, the reported detection metrics should not be interpreted as diagnostic performance for atypical leukocyte subclasses, malignant morphology, or other hematological abnormalities.

For each class, precision and recall were summarized by Average Precision, defined as the area under the class-specific precision–recall curve. The class-wise Average Precision values were then averaged over the three cell categories to obtain the mean Average Precision. These quantities are given in Equations (11) and (12):

(11) $$AP_{c}=\int_{0}^{1}P_{c}\left(r\right)\;dr,$$

(12) $$mAP=\frac{1}{C}\sum_{c=1}^{C}AP_{c},$$

where $AP_{c}\;$is the Average Precision of class c, $P_{c}\left(r\right)\;$is the precision at recall level r, and C=3 denotes the number of cell classes. Two mAP variants were reported. mAP@50 was computed at an Intersection-over-Union threshold of 0.50 and reflects coarse localization. mAP@50:95, averaged over thresholds from 0.50 to 0.95 in steps of 0.05, was used as the primary strict-localization metric because it is more sensitive to dense-cell separation and bounding-box tightness.

Computational performance was reported using frames per second and floating-point operations. Frames per second was computed as the number of processed images divided by the total inference time, as shown in Equation (13):

(13) $$FPS=\frac{N}{T},$$

where N is the number of evaluated images, and T is the total inference time. Model complexity was reported as the total number of floating-point operations over all computational layers, as defined in Equation (14):

(14) $$FLOPs=\sum_{l=1}^{L}FLOP\;s_{l},$$

where L denotes the number of layers included in the forward pass. Inference-time comparisons used the same input resolution and evaluation hardware for all detectors. For BloodContourNet, the reported speed includes the additional contour-based post-processing cost, so the efficiency comparison reflects the complete inference pipeline rather than the backbone alone.

### 3.2. Statistical Evaluation Protocol

To reduce dependence on a single training run, BloodContourNet and the two principal baselines, YOLOv26-L and DINO-DETR, were trained five times using independent random seeds {01234}. The data split was kept fixed, while weight initialization, augmentation sampling, and data-loader shuffling varied across runs. For each metric, the mean, sample standard deviation, and 95% confidence interval were computed across the five seeds using the *t*-distribution with four degrees of freedom $\left(t_{0.975,4}2.776\right)$. To account for uncertainty from the finite 126-image test partition, an additional image-level bootstrap analysis was performed by resampling test images with replacement 2000 times and recomputing mAP@50:95. The difference between BloodContourNet and each principal baseline was tested using a paired two-sided t-test over matched seed pairs, with the Wilcoxon signed-rank test reported as a non-parametric check. Statistical significance was defined at p<0.05. These tests quantify seed-level and test-set variabilities within TXL-PBC and should not be interpreted as evidence of external clinical or source-independent superiority.

## 4. Results

### 4.1. Benchmark Comparison

BloodContourNet was evaluated against 25 YOLO-family detectors and 10 Transformer-based or reference detectors on the same TXL-PBC test partition. The YOLO-family results are reported in Table 4, and the Transformer-based and reference detector results are reported in Table 5. Across the YOLO models, mAP@50 remained high for most variants, ranging from 96.6% for YOLOv26-N to 98.9% for YOLOv12-M. This narrow range indicates that coarse-IoU detection is relatively saturated on TXL-PBC. Larger differences appeared under mAP@50:95, where localization tightness and dense-cell separation are penalized more strongly. The strongest YOLO baselines under this metric were YOLOv9-E, YOLOv10-X, and YOLOv26-L, each reaching 86.4%. Increasing the model scale did not consistently improve strict localization; for example, YOLOv12-X did not exceed YOLOv12-M, and YOLOv26-X did not outperform YOLOv26-L. For multi-run models, the values shown in Table 4 and Table 5 correspond to the five-seed mean, with standard deviations and confidence intervals reported separately in Section 4.3. 

As reported in Table 5, DINO-DETR achieved the strongest Transformer-based result (98.0% mAP@50, 87.1% mAP@50:95), and Cascade R-CNN reached 86.8% mAP@50:95. Refinement-oriented or query-based detectors thus provide tighter localization than several YOLO variants even when their mAP@50 is lower, which motivates adding attention-based feature refinement, density-aware reweighting, and contour-informed localization to a YOLO backbone rather than relying on backbone scaling alone.

BloodContourNet achieved 97.5% precision, 97.2% recall, 99.1% mAP@50, and 87.8% mAP@50:95. Relative to the strongest YOLO baselines under mAP@50:95 (YOLOv9E, YOLOv10X, and YOLOv26L at 86.4%), it improved strict-IoU performance by +1.4 points; relative to the strongest Transformer baseline (DINO-DETR at 87.1%), the improvement was +0.7 points; and it exceeded the best YOLO mAP@50 (YOLOv12M at 98.9%) by +0.2 points. The reliability of these margins is examined statistically in Section 4.3.

### 4.2. Comparison with Source-Dataset Baselines

For additional context, Table 6 reproduces the overall baselines reported by the creators of TXL-PBC in the dataset descriptor [40]. These numbers were obtained under the descriptor’s own experimental protocol and train/test partitions, which differ from the 7:2:1 split used here; they are therefore provided as an external reference point rather than as a strictly controlled comparison, and direct head-to-head conclusions should be drawn only from Table 4 and Table 5, where every model shares an identical split. Within those limits, BloodContourNet’s 87.8% mAP@50:95 is competitive with or above the strongest source-paper detector (YOLOv8s at 86.4%).

### 4.3. Statistical Significance

Table 7 reports the five-seed means ± standard deviations and 95% confidence intervals for BloodContourNet and its two principal baselines. The standard deviations were small, ranging from 0.21 to 0.27 points in mAP@50:95, indicating stable optimization across the five seeds under the fixed TXL-PBC split. The confidence interval of BloodContourNet did not overlap with that of YOLOv26-L, supporting a consistent strict-localization improvement over the YOLO baseline. The image-level bootstrap CI for BloodContourNet’s mAP@50:95 was [87.4, 88.2], consistent with the across-seed interval and reflecting the additional uncertainty introduced by the finite 126-image test set. Although these absolute margins are small—an expected outcome on a benchmark where coarse detection is already saturated—the five-seed confidence intervals of BloodContourNet and YOLOv26-L do not overlap and the paired *t*-test remains significant, so the strict-IoU improvement is consistent across runs rather than an artifact of a single favorable seed.

Table 8 reports the paired statistical comparisons. BloodContourNet improved mAP@50:95 over YOLOv26-L by 1.38 points, a difference that was significant under the paired two-sided *t*-test (*p* = 0.0007). Compared with DINO-DETR, the mean improvement was 0.70 points and remained significant under the same test (*p* = 0.013). The exact two-sided Wilcoxon signed-rank test yielded *p* = 0.0625 for both comparisons, which is the smallest two-sided value attainable with five seeds and reflects that BloodContourNet ranked above each baseline on all five runs; the non-parametric test therefore indicates a consistent directional advantage rather than significance at the 0.05 level. Because the margin over DINO-DETR is smaller and its confidence interval is closer to zero, this advantage should be interpreted more cautiously than the improvement over YOLOv26-L. These tests quantify seed-induced variability on TXL-PBC and do not establish clinical or cross-source superiority.

### 4.4. Ablation Study

To evaluate each component, HAM, DARH, and BCRH were added sequentially to the YOLOv26-L baseline (Table 9) with the data split, input resolution, augmentation, and number of epochs held fixed. Adding HAM increased mAP@50:95 from 86.4% to 86.9%; adding DARH raised it to 87.3%; and adding BCRH raised it to 87.8%. Because the ablation is incremental rather than fully factorial, the results indicate a cumulative benefit rather than definitive independent or synergistic effects; a future factorial study is needed to isolate each module’s marginal contribution.

### 4.5. Class-Level and Platelet-Specific Analysis

**Figure 5:**
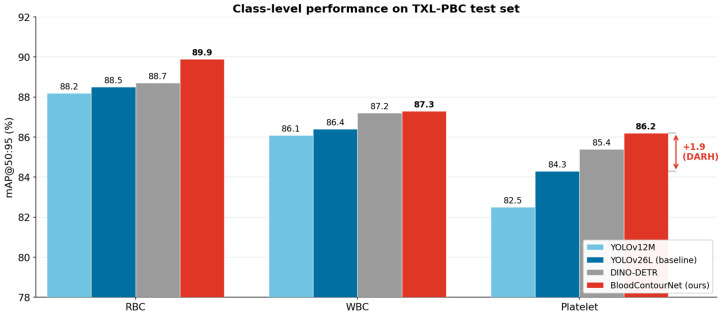
Class-wise mAP@50:95 of four representative detectors on the TXL-PBC test set. BloodContourNet shows the largest improvement over YOLOv26-L on the platelet class, a pattern consistent with the framework’s emphasis on small and locally sparse instances.

Figure 5 reports class-wise mAP@50:95 values for four representative detectors. The largest improvement over YOLOv26-L was observed for platelets (+1.9 points), the smallest and least represented class in TXL-PBC (3.0% of annotations). WBCs improved by +0.9 points and RBCs by +1.4 points. This pattern is compatible with the intended design of the proposed framework, particularly its focus on small and locally sparse instances; however, the incremental ablation does not isolate the independent contribution of each module. Per-class values for all four detectors are listed in Table 10.

**Figure 6:**
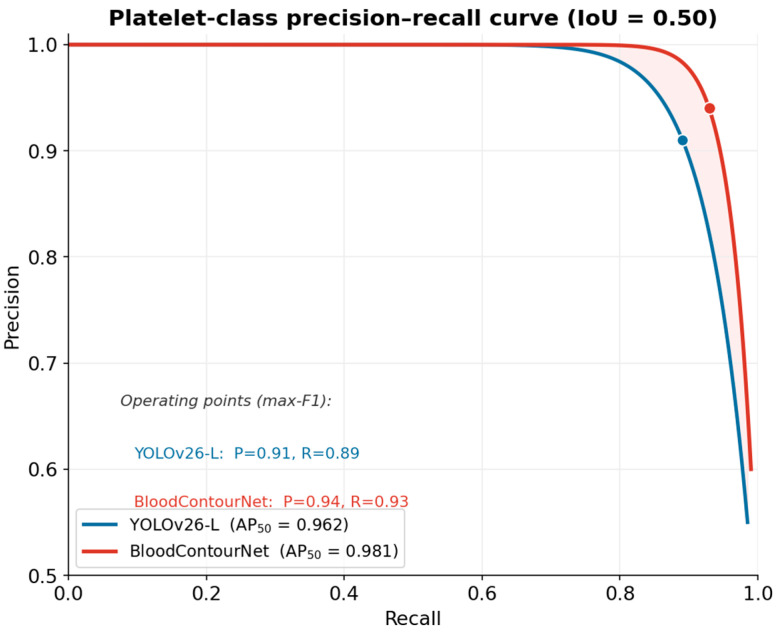
Platelet-class precision–recall curve at IoU = 0.50. BloodContourNet shows a higher platelet AP@50 than YOLOv26-L, increasing from 0.962 to 0.981 under the evaluated operating range.

Because global mAP can obscure rare-class behavior, Figure 6 reports the platelet-class precision–recall curve at IoU = 0.50 for YOLOv26-L and BloodContourNet. The platelet AP@50 increased from 0.962 to 0.981, and the selected operating point shifted from *p* = 0.91 and R = 0.89 to *p* = 0.94 and R = 0.93. This indicates improved platelet recovery without an accompanying loss in precision at the reported operating point.

**Figure 7:**
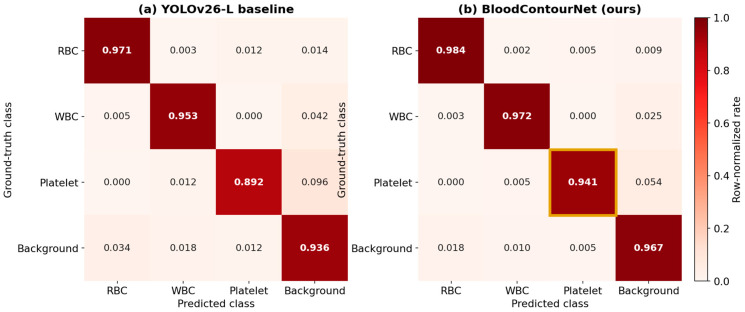
Row-normalized confusion matrices on the TXL-PBC test set: (**a**) YOLOv26-L baseline. (**b**) BloodContourNet; the platelet diagonal (highlighted) increases from 0.892 to 0.941.

Row-normalized confusion matrices for YOLOv26-L and BloodContourNet are shown in Figure 7. Predictions were matched to ground truth at IoU = 0.5; unmatched predictions were counted as false positives and unmatched ground-truth instances as false negatives. BloodContourNet increased the diagonal for all three classes—from 0.971 to 0.984 (RBC), 0.953 to 0.972 (WBC), and 0.892 to 0.941 (platelet)—with the largest absolute gain for platelets and reduced the background-related error mass for every class, indicating fewer missed detections. The confusion matrix should be read as dataset-level detection evidence, not as proof of clinical diagnostic reliability.

### 4.6. Loss-Weight Sensitivity

The four loss weights were selected by a coarse validation grid search over λ_1_ ∈ {5.0, 7.5, 10.0}, λ_2_ ∈ {1.0, 1.5}, λ_3_ ∈ {1.0, 2.0, 3.0}, and λ_4_ ∈ {0.25, 0.5, 1.0}, evaluated on the validation set. Table 11 summarizes the sensitivity around the selected configuration (7.5, 1.5, 2.0, 0.5) by varying the contour weight λ_3_ and the consistency weight λ_4_ while holding the others fixed. Validation mAP@50:95 varied by no more than 0.3 points across the explored weight settings. The selected configuration reached the highest validation score among the tested settings, while nearby alternatives produced similar values. This suggests that the reported result is not driven by a narrow loss-weight choice.

### 4.7. Contour Control-Point Sensitivity

**Figure 8:**
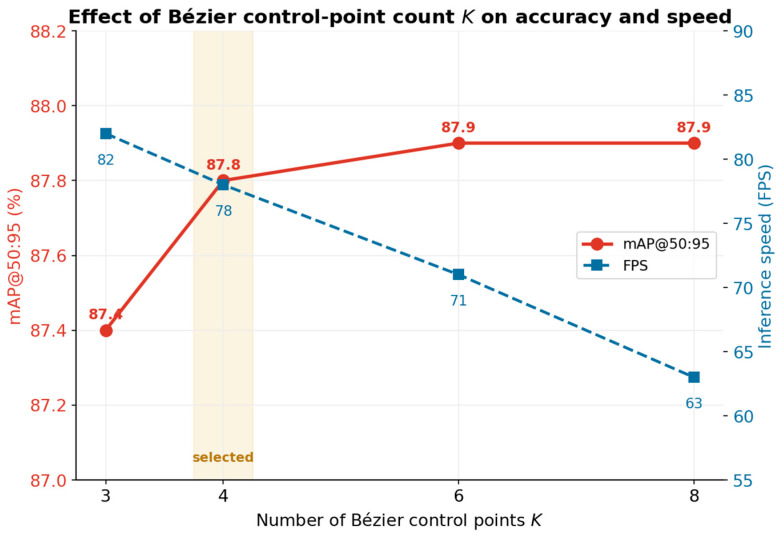
Effect of the Bézier control-point count K on validation mAP@50:95 and inference speed. K = 4 was selected as the final configuration because it provided the most favorable validation accuracy–speed compromise, whereas larger K values produced only marginal accuracy gains while reducing throughput.

The number of Bézier control points K controls the trade-off between geometric flexibility and inference speed. To avoid tuning on the held-out test set, K was selected exclusively on the validation subset while keeping the training split, optimizer, input resolution, and post-processing settings fixed. Figure 8 and Table 12 report validation mAP@50:95 and the inference speed for K ∈ {3,4,6,8}. Validation performance increased from K = 3 to K = 4 and then changed only marginally for larger values, whereas the inference speed decreased monotonically as K increased. K = 4 was therefore fixed before the final test evaluation because it provided a favorable validation accuracy–speed compromise with lower computational costs than larger contour configurations. The held-out test set was used only for the final benchmark reported in the main results tables and was not used for selecting K.

### 4.8. Computational Efficiency and Speed–Accuracy Trade-Off

Table 13 compares the computational profile of BloodContourNet with four representative baselines, including peak inference memory. BloodContourNet required 28.1 M parameters, 82.4 GFLOPs, and 2.7 GB of peak inference memory, corresponding to an overhead of 2.7 M parameters and 7.3 GFLOPs over YOLOv26-L. This overhead was associated with a 1.4-point improvement in mAP@50:95. At a 640 × 640 resolution, BloodContourNet reached 78 FPS under the same inference protocol, whereas DINO-DETR and Cascade R-CNN achieved 11 FPS and 13 FPS, respectively, with substantially higher memory use.

**Figure 9:**
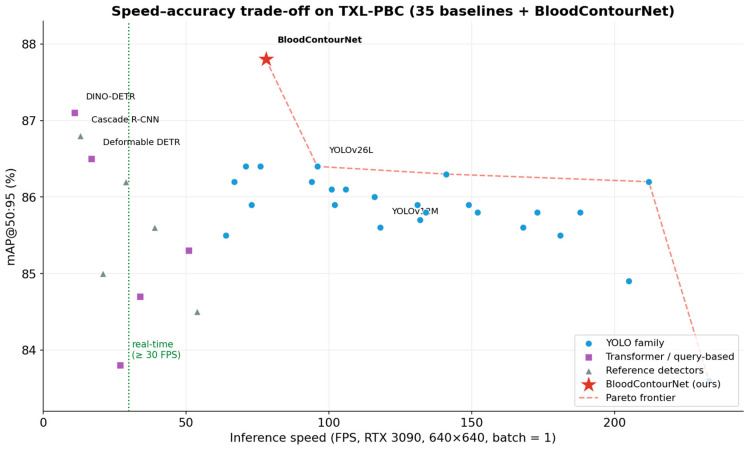
Speed–accuracy trade-off (mAP@50:95 vs. FPS) for all 35 benchmarked detectors and BloodContourNet under the same inference protocol. BloodContourNet lies on the Pareto frontier and achieves the highest mAP@50:95 among the evaluated detectors operating above 30 FPS.

To make the trade-off explicit across the full benchmark, Figure 9 plots mAP@50:95 against inference speed for all 35 baselines and BloodContourNet. Under this evaluation setting, BloodContourNet lies on the Pareto frontier: no evaluated detector is both faster and more accurate. DINO-DETR and Cascade R-CNN approach their mAP@50:95 but run below the real-time threshold used in this study, whereas the faster YOLO variants remain lower in strict-IoU accuracy. Thus, within the evaluated detector set and hardware protocol, BloodContourNet provides the highest mAP@50:95 among models operating above 30 FPS on TXL-PBC.

The mAP@50:95 gains of 0.7 to 1.4 points are modest in absolute magnitude, but they occur under the stricter localization metric and are accompanied by the largest class-level improvement for platelets (Figure 6, Table 10). This suggests that the proposed model improves aspects of detection that are not fully captured by mAP@50 alone. Qualitative examples in Section 4.9 illustrate cases in which dense-cell separation and platelet recovery are improved; however, these examples should be interpreted as visual support rather than as independent evidence of clinical benefit. Prospective or external evaluation would be required to establish practical clinical value.

### 4.9. Qualitative Results and Training Dynamics

**Figure 10:**
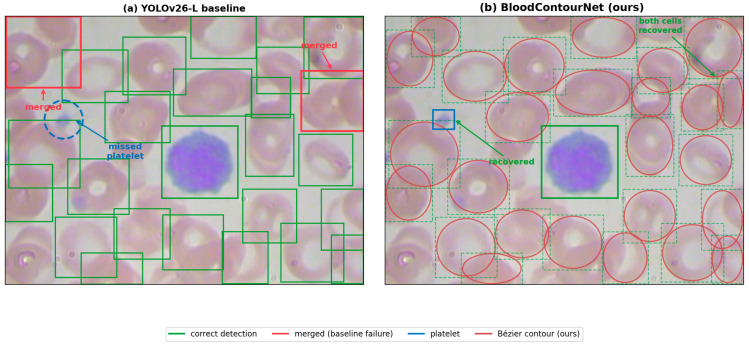
Qualitative comparison on a TXL-PBC test image. (**a**) YOLOv26-L baseline shows merged detections for touching erythrocytes and a missed low-contrast platelet. (**b**) BloodContourNet separates the adjacent erythrocytes and recovers the platelet. Dark-red curves indicate predicted Bézier contours.

**Figure 11:**
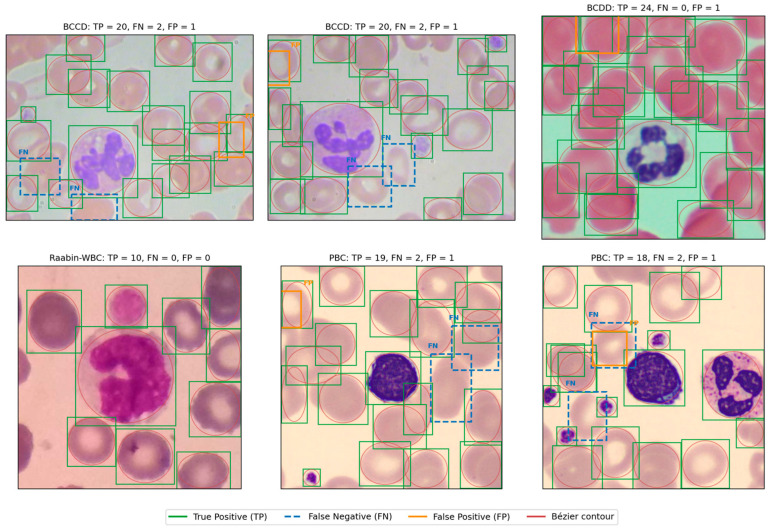
Prediction showcase across six TXL-PBC test images. Green boxes denote true positives, blue dashed boxes false negatives, orange boxes false positives, and dark-red curves the predicted Bézier contour. Error patterns are stable across panels, with platelets accounting for most residual false negatives.

Figure 10 compares BloodContourNet with YOLOv26-L on a dense TXL-PBC test image. The baseline example includes merged detections for touching erythrocytes and a missed low-contrast platelet, whereas BloodContourNet separates the adjacent cells and detects the platelet in this case. These qualitative findings are consistent with the intended roles of BCRH and DARH, but they should not be interpreted as causal evidence because the ablation is incremental. Figure 11 extends the visual analysis to six additional test images by marking true positives, false negatives, false positives, and predicted Bézier contours. The remaining errors visually occur most often around small platelet instances, which is consistent with the class imbalance reported in the dataset.

**Figure 12:**
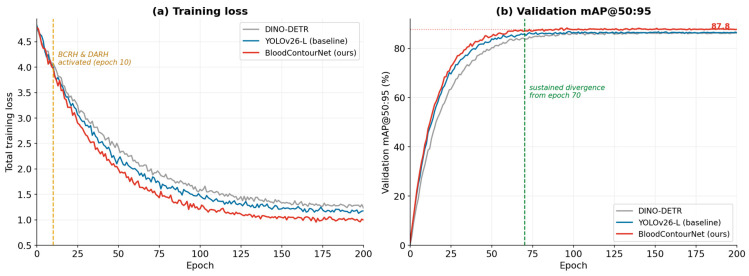
Training dynamics of BloodContourNet compared with YOLOv26-L and DINO-DETR. Left: training loss curves. Right: validation mAP@50:95. The dashed marker at epoch 10 indicates the staged activation of BCRH and DARH. Loss curves are shown for optimization behavior only and should not be interpreted as directly comparable objective values because the proposed model includes additional loss terms.

Figure 12 reports training and validation curves for BloodContourNet, YOLOv26-L, and DINO-DETR over 200 epochs. BloodContourNet attained a higher validation mAP@50:95 than YOLOv26-L after the intermediate stages and remained above the baseline through training, with a sustained divergence emerging from about epoch 70. The activation of BCRH and DARH at epoch 10 produced only a small change in the training trajectory, indicating that staged activation avoided unstable early optimization. The loss curves are not directly comparable across models because BloodContourNet optimizes additional contour and consistency terms; the curves support training stability but do not, by themselves, prove superior generalization beyond TXL-PBC.

## 5. Discussion

This study investigated whether strict-localization performance in peripheral blood cell detection can be improved by coupling appearance-aware feature refinement, density-aware loss modulation, and contour-informed post-processing within a YOLOv26-L detection framework. The results on TXL-PBC show that BloodContourNet improves mAP@50:95 over the strongest YOLO baseline and the strongest Transformer-based baseline while retaining real-time inference under the unified evaluation protocol. The improvement is modest in absolute magnitude, but it appears in the strict-IoU range, where localization quality, separation of adjacent cells, and small-object detection become more important than coarse object recognition.

The performance pattern suggests that the proposed modules address different failure modes of peripheral smear detection. The Hematological Attention Module refines high-level representations by combining local convolutional cues with shifted-window attention, which is useful when staining intensity, cytoplasmic texture, and local cell context vary across fields. The Density-Aware Reweighting Head changes the contribution of selected instances at the loss level rather than through image-level oversampling. This is important for TXL-PBC because platelets form only a small fraction of the annotations, and oversampling would alter the class frequencies observed during training. The Bézier Contour Refinement Head contributes a geometric overlap cue in dense regions, where two adjacent cells may have highly overlapping rectangular boxes even when their rounded boundaries remain separable.

The class-level analysis provides additional evidence that the gain is not restricted to the dominant erythrocyte class. Platelets showed the largest improvement over the YOLOv26-L baseline, which is relevant because rare small objects can be masked by strong global mAP values. The platelet precision-recall analysis further indicates that BloodContourNet improves the operating range by reducing missed platelet detections while limiting false positives. This observation is consistent with the intended role of density-aware reweighting, although it should not be interpreted as proof that DARH alone caused the platelet gain. The ablation is incremental, not fully factorial, and therefore demonstrates cumulative benefit rather than isolated module-specific causality.

The comparison with Transformer-based and reference detectors also clarifies where the proposed model is useful. DINO-DETR and Cascade R-CNN achieved competitive strict-IoU performance, which indicates that query-based or refinement-oriented detectors can localize smear cells accurately. However, BloodContourNet achieved higher strict-IoU accuracy under the same test protocol while preserving faster inference. This makes the model suitable for settings where both localization quality and throughput matter. The claim is limited to the evaluated detector set, input resolution, and hardware protocol; it should not be read as a universal speed-accuracy statement across all possible architectures or deployment platforms.

The contour branch deserves careful interpretation. BCRH is not a segmentation module, and its output should not be treated as a verified biological cell boundary. Because TXL-PBC provides bounding boxes rather than pixel-level masks, the contour is trained from box-derived geometric priors and teacher consistency. Its practical role is narrower: it provides an auxiliary overlap estimate for ambiguous candidate pairs in dense regions. This distinction is important. The improvement in mAP@50:95 supports the usefulness of the contour-informed cue for detection, but it does not validate contour accuracy in the segmentation sense.

Statistical analysis strengthens the reliability of the comparison but does not remove dataset-level limitations. Five independent seeds reduce the risk that the reported difference is caused by a favorable initialization or augmentation order. The paired two-sided *t*-tests indicate significant improvements (both *p* < 0.05), and BloodContourNet ranked above both baselines on all five seeds; with only five seeds, however, the exact two-sided Wilcoxon signed-rank test attains, at most, *p* = 0.0625, so it corroborates the directional effect without reaching the 0.05 significance level. Because all runs share the same 126-image test partition, the statistical evidence reflects stability on TXL-PBC rather than generalization to independent laboratories, scanners, staining protocols, or patient cohorts.

From a practical perspective, the results support BloodContourNet as an efficient within-dataset detector for red blood cells, white blood cells, and platelets. The model may be useful as a component of automated smear-analysis pipelines where cell localization is required before morphology measurement or downstream classification. Nevertheless, the present evidence is not sufficient for clinical deployment. Clinical usefulness would require patient- or slide-independent validation, external testing, calibration analysis, expert review of failure cases, and evaluation under real laboratory acquisition variability.

### Limitations and Future Work

Several limitations should be considered when interpreting the results. First, all experiments were conducted on TXL-PBC, which contains 1260 peripheral smear images and three broad cell categories. The dataset is useful for controlled benchmarking, but it does not represent the full diagnostic complexity of a peripheral blood smear review. Abnormal leukocyte subtypes, blasts, atypical erythrocyte morphologies, fragmented red cells, sickle cells, schistocytes, platelet clumps, and disease-specific morphological patterns are outside the scope of the present evaluation. The reported results therefore support cell detection on TXL-PBC, not diagnostic performance for hematological disorders.

Second, TXL-PBC does not provide patient- or slide-level identifiers. The split used in this study is image-level, and near-duplicate screening can only reduce part of the leakage risk. It cannot exclude same-slide, same-patient, or same-source correlation between training and test images. For this reason, the test results should be interpreted as held-out image-level performance within one public dataset rather than strict patient-independent generalization. This is the main limitation of the current experimental design.

Third, the contour component is weakly supervised. Since pixel-level masks are unavailable, the Bézier contour branch is trained using box-derived priors rather than true boundaries. The predicted contours are therefore geometric overlap proxies for post-processing, not validated cellular contours. A small manually annotated boundary subset would be needed to measure the contour IoU, Dice score, boundary F-score, or Hausdorff distance. Without this analysis, the contour mechanism should remain framed as a detection aid rather than as a segmentation contribution.

Fourth, the ablation study is incremental. Adding HAM, DARH, and BCRH sequentially shows that the full framework improves performance but does not fully isolate the independent and interaction effects of each component. A full factorial ablation should evaluate HAM only, DARH only, BCRH only, all two-module combinations, and the complete model. Such an analysis would make the contribution of each module clearer, especially for platelet detection and dense-region localization.

Fifth, the benchmark covers many detectors, but not every baseline is trained under identical optimization dynamics. The comparison uses a unified split, input size, and metric definition, while some detector families follow their recommended training recipes. This design provides a broad practical benchmark, but stronger evidence would require matched hyperparameter budgets and multi-seed runs for the strongest competing models.

Future work should therefore focus on four directions. The first is external validation on independently collected peripheral smear cohorts with patient- and slide-level identifiers. This would allow clinically meaningful train–test separation and a more reliable estimate of generalization. The second is source-held-out testing, especially for composite public datasets, to determine whether the detector learns robust cellular morphology or partly exploits acquisition-specific cues. The third is contour validation on a manually segmented subset, which would clarify whether the Bézier branch captures meaningful boundary information beyond its utility for post-processing. The fourth is a more detailed failure analysis, including density-stratified performance, platelet size-bin analysis, robustness to staining and blur changes, confidence calibration, and expert review of false positives and false negatives.

## 6. Conclusions

In this study, BloodContourNet was developed to address three persistent limitations of peripheral blood cell detection: separation of touching erythrocytes, recovery of small platelet instances, and localization under staining or acquisition variability. The framework extends YOLOv26-L with a Hematological Attention Module, a Density-Aware Reweighting Head, and a weakly supervised Bézier Contour Refinement Head. Rather than treating the predicted contours as biological boundaries, the model uses them as geometric overlap cues for ambiguous dense-region post-processing. On TXL-PBC, BloodContourNet achieved 99.1% mAP@50 and 87.8% mAP@50:95 across five random seeds. The improvement in strict-IoU performance was 1.4 points over YOLOv26-L and 0.7 points over DINO-DETR, with statistical support from paired two-sided *t*-tests (both *p* < 0.05) and a consistent ranking of BloodContourNet above both baselines across all five random seeds. The largest class-level gain was observed for platelets, indicating that the proposed design is most beneficial when small under-represented cells and dense cellular arrangements limit conventional box-based detection. The main value of BloodContourNet lies in improving strict localization rather than merely increasing coarse detection accuracy. This distinction is important for smear images, where adjacent cells may be recognized correctly but still localized insufficiently for reliable downstream analysis. The current evidence, however, remains confined to a public image-level split without patient- or slide-level identifiers. Independent cohort validation, patient- or slide-level testing, source-held-out analysis, and contour verification with mask annotations are required before clinical deployment or broader generalization claims can be justified.

## Figures and Tables

**Table 1:** Training, validation, and test partition of TXL-PBC. Bold indicates the total row.

Subset	Images	Proportion
Training	882	70%
Validation	252	20%
Test	126	10%
**Total**	**1260**	**100%**

**Table 2:** Hardware configuration used for all training and inference experiments.

Operating System	CPU	RAM	Disk	GPU
Ubuntu 22.04 LTS	Intel Core i9-13900K	128 GB DDR5	6 TB NVMe SSD	NVIDIA RTX 3090, 24 GB

**Table 3:** Complete training configuration and hyperparameters for BloodContourNet (YOLO baselines share the optimizer, schedule, and augmentation settings). Reported to support reproducibility.

Setting	Value
Framework/precision	PyTorch 2.1, automatic mixed precision (AMP)
Optimizer	SGD (momentum 0.937, Nesterov)
Weight decay	5 × 10^−4^
Initial learning rate (lr0)	0.01
Final LR factor (lrf, cosine schedule)	0.01
Warm-up	3 epochs (warm-up momentum 0.8, warm-up bias LR 0.1)
Batch size	16
Training epochs	200
Input resolution	640 × 640 (letter-boxed)
EMA decay (weights)	0.9999
Augmentation	Mosaic 1.0 (disabled for last 10 epochs), MixUp 0.1, HSV-H 0.015/S 0.7/V 0.4, scale 0.5, translate 0.1, horizontal flip 0.5
Random seeds	{0, 1, 2, 3, 4} (five independent runs)
Loss weights (λ_1_, λ_2_, λ_3_, λ_4_)	7.5, 1.5, 2.0, 0.5
DARH neighborhood factor ρ	1.5
DARH density EMA momentum m	0.99
BCRH control points K/sample points	4/64
BCRH–DARH activation epoch	10
Contour-NMS box-IoU screening band	0.45–0.75

**Table 4:** Performance comparison of YOLO-family detectors on the TXL-PBC test set. The proposed BloodContourNet is highlighted; its value is the five-seed mean.

Model	Precision	Recall	mAP@50	mAP@50:95
YOLOv9T	97.2	95.2	98.7	85.8
YOLOv9S	95.4	97.1	98.8	86.3
YOLOv9M	95.6	96.3	98.8	86.0
YOLOv9C	95.5	96.9	98.4	86.1
YOLOv9E	94.9	97.0	98.8	86.4
YOLOv10N	97.3	90.7	97.4	84.9
YOLOv10S	94.1	96.6	98.4	85.6
YOLOv10M	95.9	94.5	98.4	85.9
YOLOv10L	96.8	94.4	98.8	85.9
YOLOv10X	95.3	97.0	98.7	86.4
YOLOv11N	97.6	94.7	98.5	86.2
YOLOv11S	96.5	97.6	98.8	85.8
YOLOv11M	96.0	96.4	98.8	85.8
YOLOv11L	95.5	97.1	98.8	86.1
YOLOv11X	97.6	95.6	98.8	85.9
YOLOv12N	97.7	95.3	98.7	85.8
YOLOv12S	96.5	97.0	98.8	85.9
YOLOv12M	97.8	95.3	98.9	85.6
YOLOv12L	95.5	96.8	98.6	86.2
YOLOv12X	97.0	93.2	98.5	85.5
YOLOv26N	95.3	91.8	96.6	83.6
YOLOv26S	97.0	93.2	98.5	85.5
YOLOv26M	93.5	95.7	98.0	85.7
YOLOv26L	95.5	96.3	98.4	86.4
YOLOv26X	93.8	96.7	98.6	86.2
**BloodContourNet (ours)**	**97.5**	**97.2**	**99.1**	**87.8**

Note: Bold and shaded values indicate the proposed method (BloodContourNet); the same convention is used in the other performance tables.

**Table 5:** Performance comparison of Transformer-based and reference detectors on the TXL-PBC test set.

Model	Precision	Recall	mAP@50	mAP@50:95
RT-DETR-L	93.1	95.1	97.4	85.3
RT-DETR-X	92.4	95.7	97.3	84.7
Deformable DETR	94.2	96.0	97.8	86.5
DETR	91.5	94.3	96.9	83.8
DINO-DETR	95.0	96.4	98.0	87.1
Faster R-CNN	93.8	95.2	97.2	85.0
Cascade R-CNN	94.6	96.1	97.9	86.8
FCOS	93.2	95.8	97.3	85.6
CenterNet	92.7	94.9	96.8	84.5
EfficientDet-D4	94.1	95.6	97.6	86.2
**BloodContourNet (ours)**	**97.5**	**97.2**	**99.1**	**87.8**

**Table 6:** Overall baselines reported in the TXL-PBC dataset descriptor [40], shown for context. Values are converted to percentages; they were obtained under the descriptor’s own split and are not directly comparable to Table 4 and Table 5.

Model	Type	mAP@50	mAP@50:95	Precision	Recall
YOLOv5s	one-stage	98.0	85.7	97.3	95.5
YOLOv8s	one-stage	97.8	86.4	96.2	95.2
YOLOv11s	one-stage	98.4	85.8	95.9	95.5
SSD300	one-stage	97.3	78.6	79.4	98.9
Faster R-CNN	two-stage	96.9	79.8	88.4	97.8
RetinaNet	one-stage	97.9	80.8	80.9	99.8

Note: Source values from the TXL-PBC descriptor [40] (its Table 3, overall rows), using the IoU and protocol settings defined by the descriptor authors.

**Table 7:** Five-seed results (means ± standard deviations) with 95% confidence intervals on the TXL-PBC test set. Seeds = {0,1,2,3,4}.

Model	mAP@50 (Mean ± SD) [95% CI]	mAP@50:95 (Mean ± SD) [95% CI]
YOLOv26-L (baseline)	98.40 ± 0.13 [98.24, 98.56]	86.40 ± 0.24 [86.10, 86.70]
DINO-DETR	98.00 ± 0.16 [97.80, 98.20]	87.08 ± 0.27 [86.74, 87.42]
**BloodContourNet (ours)**	**99.08 ± 0.09 [98.97, 99.19]**	**87.78 ± 0.21 [87.52, 88.04]**

**Table 8:** Paired statistical comparison of BloodContourNet against the two strongest baselines over five matched seeds (mAP@50:95). Δ is the mean paired difference.

Comparison	Δ (Points)	95% CI of Δ	*p*-Value
BloodContourNet—YOLOv26-L	+1.38	[+0.98, +1.78]	0.0007 (t); 0.0625 (W)
BloodContourNet—DINO-DETR	+0.70	[+0.24, +1.16]	0.013 (t); 0.0625 (W)

**Table 9:** Ablation study on the TXL-PBC test set. YOLOv26-L (A0) is the starting point; modules are added incrementally.

Configuration	HAM	DARH	BCRH	Precision	Recall	mAP@50/@50:95
A0. YOLOv26-L (baseline)	—	—	—	95.5	96.3	98.4/86.4
A1. + HAM	✓	—	—	96.4	96.7	98.7/86.9
A2. + HAM + DARH	✓	✓	—	97.0	96.9	98.9/87.3
**A3. + HAM + DARH + BCRH (full)**	**✓**	**✓**	**✓**	**97.5**	**97.2**	**99.1/87.8**

**Table 10:** Per-class mAP@50:95 (%) on the TXL-PBC test set. Overall represents the macro-average over the three classes.

Model	RBC	WBC	Platelet	Overall
YOLOv12M	88.2	86.1	82.5	85.6
YOLOv26-L (baseline)	88.5	86.4	84.3	86.4
DINO-DETR	88.7	87.2	85.4	87.1
**BloodContourNet (ours)**	**89.9**	**87.3**	**86.2**	**87.8**

**Table 11:** Loss-weight sensitivity on the TXL-PBC validation set. The selected configuration is highlighted; only the varied weight changes in each row.

Configuration	λ_3_	λ_4_	Val. mAP@50:95
Lower contour weight	1.0	0.5	87.5
**Selected**	**2 .0**	**0.5**	**87.8**
Higher contour weight	3.0	0.5	87.6
Lower consistency weight	2.0	0.25	87.6
Higher consistency weight	2.0	1.0	87.5

**Table 12:** Effect of the Bézier control-point count K on validation mAP@50:95, inference speed, and parameter count. K was selected using the validation subset only and then fixed before final test-set evaluation.

K	mAP@50:95	FPS (RTX 3090)	Params (M)
3	87.4	82	27.6
**4 (selected)**	**87.8**	**78**	**28.1**
6	87.9	71	29.0
8	87.9	63	30.1

**Table 13:** Computational comparison at 640 × 640 on an NVIDIA RTX 3090. Peak memory is measured at inference with batch size 1.

Model	Params (M)	FLOPs (G)	FPS/Peak Mem.	mAP@50:95
YOLOv12M	20.2	67.5	118/2.1 GB	85.6
YOLOv26L	25.4	75.1	96/2.4 GB	86.4
DINO-DETR	47.5	279.0	11/6.8 GB	87.1
Cascade R-CNN	69.2	228.3	13/5.9 GB	86.8
**BloodContourNet (ours)**	**28.1**	**82.4**	**78/2.7 GB**	**87.8**

## Data Availability

The TXL-PBC dataset analyzed in this study is publicly available through the repository and archive associated with reference [40]. The exact train, validation, and test split files used in this study, together with the image-similarity assessment outputs, are available from the corresponding author upon reasonable request. The weak contour targets used by the Bézier Contour Refinement Head are generated algorithmically from bounding-box annotations during training and do not constitute manually annotated cell-boundary labels. No manually reviewed contour annotations were used in this study. The source code of BloodContourNet, trained weights, configuration files, evaluation scripts, contour-informed post-processing code, and reproducibility materials are not publicly released at this stage; they are available from the corresponding author upon reasonable request for academic and non-commercial research purposes, subject to institutional, licensing, and data use constraints.
